# The breaking point in robotic pancreaticoduodenectomy: factors influencing conversion thresholds and early postoperative outcomes in a tertiary referral center

**DOI:** 10.1007/s00464-026-12805-6

**Published:** 2026-04-22

**Authors:** Alessia Fassari, Edouard Wasielewski, Antoine Castel, Hector Prudhomme, Salaheddine Abdennebi, Aude Merdrignac, Marie Livin, Fabien Robin, Laurent Sulpice

**Affiliations:** 1 Hepatobiliary and Digestive Surgery Department, University Hospital of Rennes, Rennes, France; 2 French Institute of Health and Medical Research | Inserm U1414, University Hospital of Rennes, Rennes, France; 3 Oncogenesis Stress Signalling (OSS), Inserm U1242, OSS, University Hospital of Rennes, Rennes, France; 4 Centre Hospitalier Universitaire de Rennes, 2, rue Henri le Guilloux, 35000 Rennes, France

**Keywords:** Robotic pancreatic surgery, Pancreaticoduodenectomy, Surgical conversion, Predictive factors, Postoperative outcomes

## Abstract

**Background:**

Robotic pancreaticoduodenectomy (RPD) has expanded the indications of minimally invasive pancreatic surgery. However, conversion to open surgery remains a relevant intraoperative event, and data on its determinants and clinical impact in real-world robotic programs are limited. Conversion is increasingly regarded as a safety-driven decision rather than a technical failure, particularly in experienced centers.

**Methods:**

This retrospective single-center cohort study included all consecutive adult patients undergoing intended RPD between April 2018 and October 2025. Conversion was defined as any unplanned laparotomy after initiation of the robotic approach. Patient-, disease-, and procedure-related variables were analyzed. Factors associated with conversion were explored using univariable analyses and multivariable Firth penalized logistic regression. Postoperative outcomes were assessed descriptively according to conversion status using effect size measures.

**Results:**

Among 130 patients undergoing RPD, 16 (12.3%) required conversion. On multivariable analysis, vascular contact requiring resection emerged as the strongest factor associated with conversion, followed by periampullary tumor location, while previous pancreatitis showed a borderline association. Most conversions occurred early in the procedure (75%) and were strategic rather than urgent (87.5%). Conversion was associated with higher intraoperative transfusion rates and increased postoperative resource utilization, including longer hospital and high-dependency unit stay. No clear evidence of a large increase in major postoperative morbidity was observed, and rates of major postoperative complications, pancreatic fistula (grade B/C), and 30-day mortality were similar between groups, although estimates remain imprecise due to the limited number of converted cases.

**Conclusions:**

In a high-volume robotic pancreatic program, conversion during RPD was primarily driven by anatomical and disease-related complexity rather than surgical inexperience. When performed in a timely and controlled manner, conversion may represent a proactive safety strategy rather than a technical failure, although these findings should be interpreted cautiously given the limited number of conversion events.

**Graphical Abstract:**

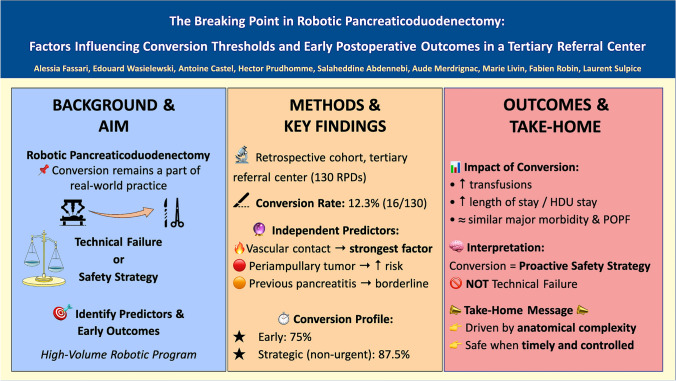

Robotic pancreaticoduodenectomy (RPD) has emerged as a major evolution of minimally invasive pancreatic surgery, offering enhanced three-dimensional visualization, wristed instrumentation, and improved surgeon ergonomics. While these technical advantages over conventional laparoscopy are increasingly recognized, the extent to which RPD confers a meaningful clinical benefit over open pancreaticoduodenectomy remains incompletely defined.

Recent high-quality comparative studies, including the DIPLOMA-2 trial, have focused on highly selected populations, restricting enrollment to upfront-resectable tumors without major vascular involvement and limiting participation to expert centers operating under strict credentialing requirements [1]. Although such trial designs ensure strong internal validity, they inevitably limit external generalizability. Consequently, the real-world safety, effectiveness, and intraoperative behavior of RPD in less selected, clinically heterogeneous populations remain to be fully characterized.

Within this context, conversion to open surgery represents a pivotal intraoperative event during RPD. Traditionally regarded as a marker of technical failure, conversion may instead reflect appropriate surgical judgment in response to bleeding, unfavorable anatomy, limited exposure, or oncologic concerns. In this setting, conversion should be viewed as the result of a dynamic intraoperative decision-making process, in which the surgeon weighs technical feasibility, oncologic adequacy, and patient safety rather than a binary technical endpoint. Several patient-, disease-, and procedure-related factors have been proposed as drivers of conversion in minimally invasive pancreatic surgery [2–4]. However, data specifically focused on RPD remain scarce. Moreover, little is known about how these factors shape conversion thresholds once the learning curve has been surpassed, or how conversion influences postoperative outcomes in mature robotic programs, where experience, case selection, and perioperative pathways have stabilized.

Against this background, the aims of the present single-center retrospective study were threefold: (1) to identify patient-, disease-, and procedure-related factors associated with conversion during RPD; (2) to determine which of these factors remained independently associated with conversion after multivariable adjustment; and (3) to characterize the early postoperative impact of conversion, with particular attention to postoperative morbidity and length of hospital stay. By addressing these objectives, this study seeks to better define the factors influencing conversion decisions and to delineate the early clinical consequences of conversion during RPD in a high-volume setting.

## Patients and methods

### Study design and setting

This retrospective single-center cohort study was designed, conducted, and reported in accordance with the Strengthening the Reporting of Observational Studies in Epidemiology (STROBE) guidelines [5]. It included all consecutive adult patients who underwent RPD at a tertiary referral center for pancreatic surgery between April 2018 and October 2025.

The institution is a dedicated center for complex oncologic surgery, with a strong focus on hepatopancreatobiliary (HPB) procedures, and is also active in liver transplantation. The center is equipped with three da Vinci robotic systems, a dedicated pancreatic surgery unit, and comprehensive perioperative facilities, including an intensive care unit (ICU) and a high-dependency unit (HDU), allowing structured postoperative management of high-risk patients. In our institution, patients undergoing pancreaticoduodenectomy are routinely admitted to the HDU in the immediate postoperative period for close monitoring of hemodynamic parameters, fluid balance, and early detection of complications such as bleeding or metabolic disturbances. Patients are typically managed in the HDU for the first 72 h postoperatively, allowing continuous monitoring during the early postoperative phase. Transfer to the surgical ward is considered once the patient is hemodynamically stable, without need for vasoactive support or advanced monitoring, according to standardized institutional criteria.

The robotic pancreatic program was established and conducted as a single-surgeon series by a dedicated pancreatic surgeon with extensive prior experience in open pancreaticoduodenectomy and formal training in minimally invasive and robotic surgery. At the start of the study period, the surgeon had already performed more than 500 pancreatic resections using open and laparoscopic approaches, providing a solid background in pancreatic surgery at the initiation of the robotic program. The present series also encompasses the early phase of the robotic learning curve. All RPDs were performed using a standardized four-arm robotic platform (da Vinci system; Intuitive Surgical, Sunnyvale, CA, USA), with a uniform port-placement strategy and consistent operative setup.

The study was conducted in accordance with the Declaration of Helsinki [6]. Institutional review board approval was obtained, and the requirement for individual informed consent was waived due to the retrospective nature of the study and full anonymization of patient data.

### Patient selection

All consecutive adult patients (≥ 18 years) who underwent elective RPD as the intended primary surgical approach for benign, premalignant, or malignant disease of the pancreatic head, distal bile duct, ampulla, or periampullary region were screened for inclusion.

At the beginning of the robotic program, patient selection was restricted to cases considered technically favorable for a minimally invasive approach, typically tumors without major vascular involvement on preoperative imaging. With increasing surgical experience and progressive integration of the robotic platform into routine clinical practice, indications were gradually expanded to include more complex cases. In particular, selected patients with borderline vascular contact at initial staging who demonstrated favorable response after neoadjuvant therapy on restaging imaging were also considered for a robotic approach, while maintaining readiness to perform venous resection if required.

Patients were eligible for inclusion if complete intraoperative and postoperative data were available to allow assessment of the primary outcome (conversion to open surgery) and secondary postoperative clinical outcomes.

Patients were excluded if a hybrid procedure was planned a priori (e.g., robotic resection with predefined open reconstruction), or if key variables required for the primary analyses, including conversion status or essential perioperative data, were missing.

### Variables and data collection

Data were extracted from a prospectively maintained institutional database and completed through targeted review of electronic medical records and operative reports.

Patient-related variables included demographic characteristics (age and sex), body mass index (BMI, analyzed both as a continuous variable and by categories), comorbidity burden as assessed by the Charlson Comorbidity Index (CCI), history of previous abdominal surgery, and documented anatomical variations when available [7].

Disease-related variables included the underlying diagnosis, tumor location and size on preoperative imaging, with tumors categorized as periampullary (including bile duct, duodenal, and ampullary tumors) or pancreatic head tumors (including pancreatic ductal adenocarcinoma and intraductal papillary mucinous neoplasms). Histology was classified as benign or malignant. Tumor stage at diagnosis was defined as upfront resectable, borderline resectable, or locally advanced based on radiologic vascular involvement (venous or arterial abutment or encasement) and biological criteria, including a serum carbohydrate antigen (CA) 19–9 level more than 500 units/ml [8]. The administration of neoadjuvant therapy was also recorded.

Surgeon- and procedure-related variables included learning-curve phase, preoperative vascular contact suggesting potential need for venous resection, main pancreatic duct diameter (≤ 3 mm), and pancreatic texture (soft vs firm).

Preoperative vascular contact was defined exclusively on the basis of high-resolution contrast-enhanced CT imaging performed at initial staging and discussed in a multidisciplinary tumor board setting. It referred to radiologic evidence of tumor-vessel contact or abutment suggesting a potential need for venous resection during surgical planning. In patients who underwent neoadjuvant therapy, vascular contact was classified according to pre-treatment imaging at diagnosis. Although post-induction radiologic reassessment could demonstrate apparent regression of vascular involvement, persistent tumor-vessel contact or dense perivascular fibrosis may still be encountered intraoperatively. Anchoring the definition to initial staging imaging therefore allowed us to capture baseline anatomical complexity relevant to operative planning, rather than relying solely on post-treatment radiologic appearance.

Conversion served as the dependent variable in the primary analyses and was defined in accordance with the Brescia consensus guidelines for minimally invasive pancreatic surgery [9]. It was considered as any unplanned transition from a robotic approach to a formal open laparotomy at any stage of the procedure, whether during resection or reconstruction. A planned mini-laparotomy performed solely for specimen extraction was not considered a conversion.

In addition to the Brescia-based definition, conversions were further characterized according to timing and clinical intent [9]. Timing was categorized as early (occurring before pancreatic transection or major vascular dissection) or late (occurring during the resection or reconstruction phase). Clinical intent was categorized as urgent, when prompted by unexpected potentially life-threatening conditions such as uncontrolled bleeding or hemodynamic instability, or strategic (non-urgent), when performed in a hemodynamically stable patient due to technical difficulty, tumor extension, dense adhesions, or inability to safely proceed robotically.

Based on a prior internal assessment of operative time and perioperative outcomes, the learning curve for RPD at our institution was defined as the first 40 consecutive procedures. Accordingly, procedures were classified into two experience-based phases: an early learning phase (cases 1–40) and a proficiency phase (cases > 40) [10]. This dichotomization was used as an independent variable in multivariable models to account for the potential impact of surgical experience on the risk of conversion.

### Primary and secondary outcomes

The primary aim of this study was to identify patient-, disease-, and surgeon- or procedure-related factors independently associated with intraoperative transition to an open approach during RPD. The primary reason for conversion was systematically recorded to distinguish between emergency and strategic conversions.

Secondary outcomes were evaluated to assess the perioperative and early postoperative clinical impact of conversion and included intraoperative bleeding needing transfusions, operative time, overall postoperative morbidity, complication severity, length of hospital stay, HDU and ICU length of stay, hospital readmission within 30 days, resection margins, 30-day and 90-day mortality.

Postoperative complications and grading were defined according to internationally accepted criteria [11]. Postoperative pancreatic fistula (POPF) was defined according to the 2016 ISGPS criteria [12]. Consequently, only Grade B and C fistulas were considered, as Grade A is currently classified as a biochemical leak. Delayed gastric emptying and postpancreatectomy hemorrhage were also defined and graded according to ISGPS criteria [13, 14]. Biliary fistula was defined according to the International Study Group of Liver Surgery (ISGLS) metrics [15].

Margin status analysis was restricted to patients with pancreatic ductal adenocarcinoma (PDAC), given the different patterns of local spread and margin interpretation among periampullary tumor types. Resection margins were assessed according to the institutional standardized pathology protocol for pancreaticoduodenectomy specimens. The specimen was inked to identify all resection margins, including the pancreatic transection margin, bile duct margin, and retroperitoneal margin. A 1 mm clearance rule was applied, with R0 defined as a tumor-free margin ≥ 1 mm and R1 defined as tumor cells present within 1 mm of any resection margin.

### Statistical analysis

All statistical analyses were performed using IBM SPSS Statistics (IBM Corp., Armonk, NY, USA) and R statistical software (R Foundation for Statistical Computing, Vienna, Austria). All tests were two-sided. Continuous variables are reported as median and interquartile range (IQR), while categorical variables are presented as counts and percentages.

To identify factors associated with conversion, patient-, disease-, and procedure-related variables were first assessed individually using univariable analyses. Continuous variables were compared using the Mann–Whitney U test, and categorical variables were compared using Fisher’s exact test, given the limited number of conversion events. Variables showing a statistically significant association with conversion at univariable analysis (*p* < 0.05) were considered as candidates for multivariable analysis.

Because of the limited number of conversion events (*n* = 16), the multivariable model was deliberately restricted to three clinically relevant variables directly related to intraoperative decision-making. A Firth penalized likelihood logistic regression was therefore used to reduce small-sample bias and obtain finite, conservative estimates. Results are reported as odds ratios (ORs) with 95% confidence intervals (CIs). Perioperative and postoperative outcomes were analyzed descriptively according to conversion status. Binary outcomes are summarized using risk difference (RD) and risk ratio (RR) with 95% confidence intervals, while continuous outcomes are summarized as median differences between groups. Given the limited number of converted cases, these analyses were considered exploratory and were not intended to support formal hypothesis testing. The extent and pattern of missing data were assessed for all collected variables. Variables with substantial missingness were excluded from multivariable analyses.

## Results

### Temporal trends and study population

**Fig. 1:**
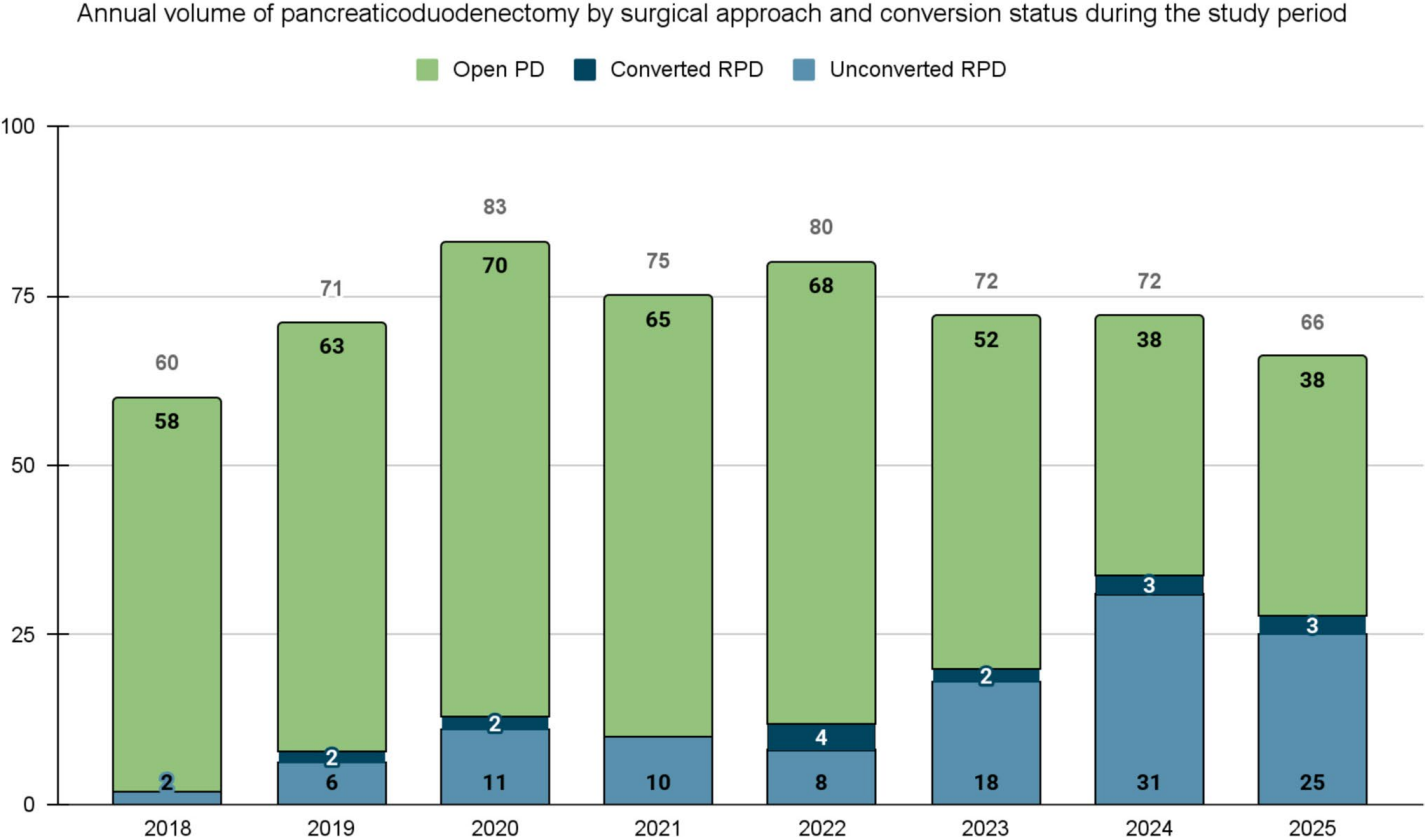
Annual volume of pancreaticoduodenectomy by surgical approach and conversion status during the study period. Annual number of pancreaticoduodenectomies performed during the study period, stratified by surgical approach. Bars display open pancreaticoduodenectomy (Open PD), completed robotic pancreaticoduodenectomy (RPD), and robotic-to-open conversions. Data for 2025 include procedures performed up to October

Figure 1 illustrates the temporal trends in pancreaticoduodenectomy volume and surgical approach during the study period. A progressive increase in the number of RPDs was observed over time, reflecting the gradual consolidation of the robotic pancreatic program. Despite this expansion in robotic case volume and the inclusion of increasingly complex cases, the number of conversions remained low and relatively stable, suggesting a controlled adoption of the robotic approach.

Overall, 130 patients underwent RPD during the study period, of whom 16 (12.3%) required conversion to an open approach. Baseline patient-, disease-, and procedure-related characteristics stratified by conversion status are summarized in Table 1.

**Table 1:** Patient, disease, and procedural characteristics stratified by conversion during RPD

Variables	Total (*n* = 130)	Unconverted RPD (*n* = 114)	Converted RPD (*n* = 16)	*P* value
Patient-related factors				
Age [median, (IQR)] (y)	67.7 (59.8–74.2)	67.2 (59.4–74.1)	72.3 (66.1–76.3)	0.10
Sex				0.28
Male [*n* (%)]	55 (42.3%)	46 (40.4%)	9 (56.3%)	
Female [*n* (%)]	75 (57.7%)	68 (59.6%)	7 (43.7%)	
CCI score				0.10
< 4 [*n*, (%)]	69 (53.1%)	64 (56.1%)	5 (31.2%)	
≥ 4 [*n*, (%)]	61 (46.9%)	50 (43.9%)	11(68.8%)	
BMI category (kg/m^2^)				0.06
< 25 [*n*, (%)]	71 (54.6%)	66 (57.9%)	5 (31.3%)	
≥ 25 [*n*, (%)]	59 (45.4%)	48 (42.1%)	11 (68.8%)	
Previous abdominal surgery [*n* (%)]	82 (63.1%)	73 (64%)	9 (56.3%)	0.59
Previous pancreatitis [*n* (%)]	15 (11.5%)	11 (9.6%)	6 (37.5%)	**0.0074**
Anatomical variations				0.59
RHA from SMA [*n*, %]	11 (8.5%)	9 (7.9%)	2 (12.5%)	
Median arcuate ligament [*n*, %]	2 (1.5%)	2 (1.8%)	0 (0%)	
CHA from SMA [*n*, %]	3 (2.3%)	3 (2.6%)	0 (0%)	
Disease-related factors				
Histology				1.00
Malignant	102 (78.5%)	89 (78%)	13 (81.3%)	
Benign	28 (21.5%)	25 (22%)	3 (18.7%)	
Tumor location				**0.004**
Periampullary [*n*, (%)]	57 (43.8%)	46 (40.4%)	11 (68.7%)	
Pancreatic (head/isthmus/uncus) [*n*, (%)]	73 (56.2%)	68 (59.6%)	5 (31.3%)	
Tumor size, [median (IQR)] (mm)	22 (15–30)	20 (15–28)	27.5 (13–38)	0.10
Stage at diagnosis				0.92
Upfront resectable [*n*, (%)]	111 (85.4%)	97 (85.1%)	14 (87.5%)	
Borderline resectable [*n*, (%)]	18 (13.8%)	16 (14%)	2 (12.5%)	
Locally advanced [*n*, (%)]	1 (0.8%)	1 (0.9%)	0 (0%)	
Neoadjuvant chemotherapy [*n*, (%)]	25 (19.2%)	23 (20.2)	2 (12.5%)	0.74
Procedure- and surgeon-related factors				
Surgeon experience^a^				0.57
Early learning phase: 1–40 procedures	40 (30.7%)	34 (29.8%)	6 (37.5%)	
Proficiency phase: > 40 procedures	90 (69.3%)	80 (70.2%)	10 (62.5%)	
Vascular contact requiring resection [*n*, %]	8 (6.2%)	1 (0.9%)	7 (43.8%)	** < 0.001**
Main pancreatic duct ≤ 3 mm [*n*, %]	60 (46.2%)	49 (43%)	11(68.8%)	0.09
Soft pancreatic texture [*n*, %]	93 (71.5%)	79 (69.2%)	14 (87.5%)	0.153

Bold values indicate statistically significant results (*p* < 0.05)

Baseline patient-, disease-, and procedure-related characteristics of patients undergoing RPD, stratified by conversion status. Continuous variables are expressed as median (IQR) and categorical variables as number (%). Variables showing a univariable association with conversion (*p* < 0.05) were considered as candidates for multivariable analysis. Given the limited number of conversion events, the multivariable model was restricted to three predictors

*RPD* Robotic pancreaticoduodenectomy, *CCI* Charlson Comorbidity Index, *BMI* body mass index; RHA right hepatic artery, *SMA* superior mesenteric artery, *CHA* common hepatic artery, *MPD* main pancreatic duct

^a^In this study we used an institution-specific cut-off of 40 based on prior internal analysis [10]

### Factors associated with conversion

On univariable analysis, three patient-, disease-, and procedure-related variables were associated with conversion to an open approach (Table 1). A history of previous pancreatitis was significantly more frequent among converted cases compared with fully robotic procedures (37.5% vs 9.6%, *p* = 0.007). Tumor location also differed between groups, with periampullary lesions being overrepresented in the converted cohort (68.7% vs 40.4%, *p* = 0.004). Among anatomical factors, vascular contact requiring resection showed the strongest univariable association with conversion and was markedly more common in converted procedures (43.8% vs 0.9%, *p* < 0.001).

Multivariable analysis using Firth penalized logistic regression is summarized in Table 2. After adjustment, vascular contact requiring resection remained the strongest factor independently associated with conversion (OR 55.0, *p* < 0.001), with wide confidence intervals reflecting the limited number of events. Periampullary tumor location also retained an independent association with conversion (OR 4.31, *p* = 0.036). Previous pancreatitis showed a borderline association after penalized adjustment (OR 5.54, *p* = 0.064).

**Table 2:** Firth penalized multivariable logistic regression for conversion

Predictors of conversion	Odds ratio (OR)	95% confidence interval	*P* value
Vascular contact requiring resection	55.04	9.23–649.14	** < 0.001**
Periampullary tumors	4.31	1.14–20.61	**0.036**
Previous pancreatitis	5.54	0.91–31.06	0.064

Bold values indicate statistically significant results (*p* < 0.05)

Multivariable analysis was performed to identify factors independently associated with conversion. Because of the limited number of conversion events (*n* = 16), a Firth penalized likelihood logistic regression was used to reduce small-sample bias and obtain finite, conservative estimates. The model included three predictors identified in univariable analysis (*p* < 0.05) and considered directly related to intraoperative decision-making. Results are reported as odds ratios (OR) with 95% confidence intervals (CI)

### Peri- and postoperative outcomes

Perioperative and postoperative outcomes according to conversion status are reported in Table 3. Given the limited number of converted cases, outcomes are presented using effect size measures to describe the direction and magnitude of differences between groups rather than to support formal hypothesis testing. Positive risk differences indicate a higher event rate among converted cases, whereas negative values indicate a lower event rate compared with fully robotic procedures. Intraoperative bleeding requiring transfusion occurred more frequently in converted procedures compared with fully robotic cases (31.3% vs 1.8%; risk difference + 29.5%). Operative time was similar between groups.

**Table 3:** Postoperative outcomes according to conversion status

Peri- and postoperative outcomes	Unconverted RPD (*n* = 114)	Converted RPD (*n* = 16)	Risk difference % (95% CI)	Risk ratio (95% CI)
Binary outcomes				
Intraoperative bleeding requiring transfusion [*n* (%)]	2 (1.8%)	5 (31.3%)	+ 29.5 (6.7 to 52.3)	17.81 (3.77–84.27)
Postoperative minor complications (CD I–II) [*n* (%)]	70 (61.4%)	9 (56.3%)	− 5.2 (− 31.1 to 20.7)	0.92 (0.58–1.45)
Postoperative major complications (CD ≥ IIIa) [*n* (%)]	28 (24.6%)	6 (37.5%)	+ 12.9 (− 12.1 to 37.9)	1.53 (0.75–3.10)
Clinically relevant DGE (B/C) [*n* (%)]	26 (22.8%)	5 (31.2%)	+ 8.4 (− 15.5 to 32.4)	1.37 (0.61–3.05)
Clinically relevant PPH (B/C) [*n* (%)]	19 (16.7%)	5 (31.2%)	+ 14.6 (− 9.1 to 38.3)	1.88 (0.81–4.32)
POPF (B/C) [*n* (%)]	15 (13.2%)	2 (12.5%)	− 0.7 (− 18.0 to 16.7)	0.95 (0.24–3.77)
Biliary Fistula [*n* (%)]	18 (15.8%)	4 (25%)	+ 9.2 (− 13.0 to 31.5)	1.58 (0.61–4.09)
Postoperative transfusion [*n* (%)]	24 (21.1%)	5 (31.2%)	+ 10.2 (− 13.7 to 34.1)	1.48 (0.66–3.33)
Reoperation [*n* (%)]	10 (8.7%)	3 (18.7%)	+ 10.0 (− 9.8 to 29.8)	2.14 (0.66–6.95)
Readmission [*n* (%)]	18 (15.8%)	4 (25%)	+ 9.2 (− 13.0 to 31.5)	1.58 (0.61–4.09)
30 day-mortality [*n* (%)]	5 (4.4%)	1 (6.2%)	+ 1.9 (− 10.6 to 14.3)	1.43 (0.18–11.43)
90 day-mortality [*n* (%)]	6 (5.3%)	2 (12.5%)	7.2% (− 9.5 to 23.9)	2.38 (0.52–10.78)
Negative resection margin (PDAC only), R0 [*n* (%)]^a^	38/42 (90.5%)	4/4 (100%)	− 9.5% (− 18.4 to − 0.6)	0.27 (0.02–4.41)

Peri- and postoperative outcomes	Unconverted RPD (*n* = 114)	Converted RPD (*n* = 16)	Median difference (converted – unconverted)	
Continuous outcomes				
Operative time [median, (IQR)] (min)	574 (546–605)	562 (550–628)	− 12	
LOS [median (IQR)] (days)	13 (9–21)	22 (14–35)	+ 9	
HDU stay [median (IQR)] (days)	7.5 (5–12)	13 (7.5–14.5)	+ 5.5	
ICU stay [median (IQR)] (days)	0 (0–0.1)	0 (0–0.75)	0	

Postoperative outcomes are compared between converted (*n* = 16) and unconverted (*n* = 114) procedures. Binary outcomes are reported as risk difference (RD) and risk ratio (RR) with 95% confidence intervals (CI), while continuous outcomes are expressed as median (interquartile range) and summarized as median difference (Converted – Unconverted). Positive RD values indicate a higher risk among converted cases. Given the limited number of conversion events, results should be interpreted as exploratory

*RPD* Robotic pancreaticoduodenectomy; *CD* Clavien-Dindo classification system, *DGE* delayed gastric emptying, *PPH* postoperative hemorrhage, *POPF* postoperative pancreatic fistula, *LOS* length of stay, *HDU* high dependency unit, *ICU* intensive care unit, *PDAC* pancreatic ductal adenocarcinoma

^a^Margin status analysis was restricted to pancreatic ductal adenocarcinoma (PDAC) to ensure comparability of oncologic margins, as other periampullary tumor types show different patterns of local spread

Postoperative bleeding-related events were more frequent among converted patients, including higher absolute rates of postoperative transfusion and reoperation. With respect to pancreas-specific outcomes, rates of POPF (grade B/C), delayed gastric emptying (grade B/C), postpancreatectomy hemorrhage, and biliary fistula were similar between converted and non-converted cases. Thirty-day postoperative mortality did not significantly differ between groups. Ninety-day mortality was numerically higher in converted cases (12.5% vs 5.3%), although this difference should be interpreted cautiously given the small number of events.

Among patients with PDAC, R0 resection was achieved in 38 of 42 non-converted cases (90.5%) and in all 4 converted cases (100%). Owing to the limited number of converted PDAC cases, these results are reported descriptively.

Conversion was associated with increased postoperative resource utilization. Median length of hospital stay was longer in converted patients (22 days, IQR 14–35) compared with non-converted patients (13 days, IQR 9–21). Similarly, median HDU stay was longer in converted cases (13 days, IQR 7.5–14.5) compared with fully robotic procedures (7.5 days, IQR 5–12).

### Reasons, timing, and intent of conversion

**Fig. 2:**
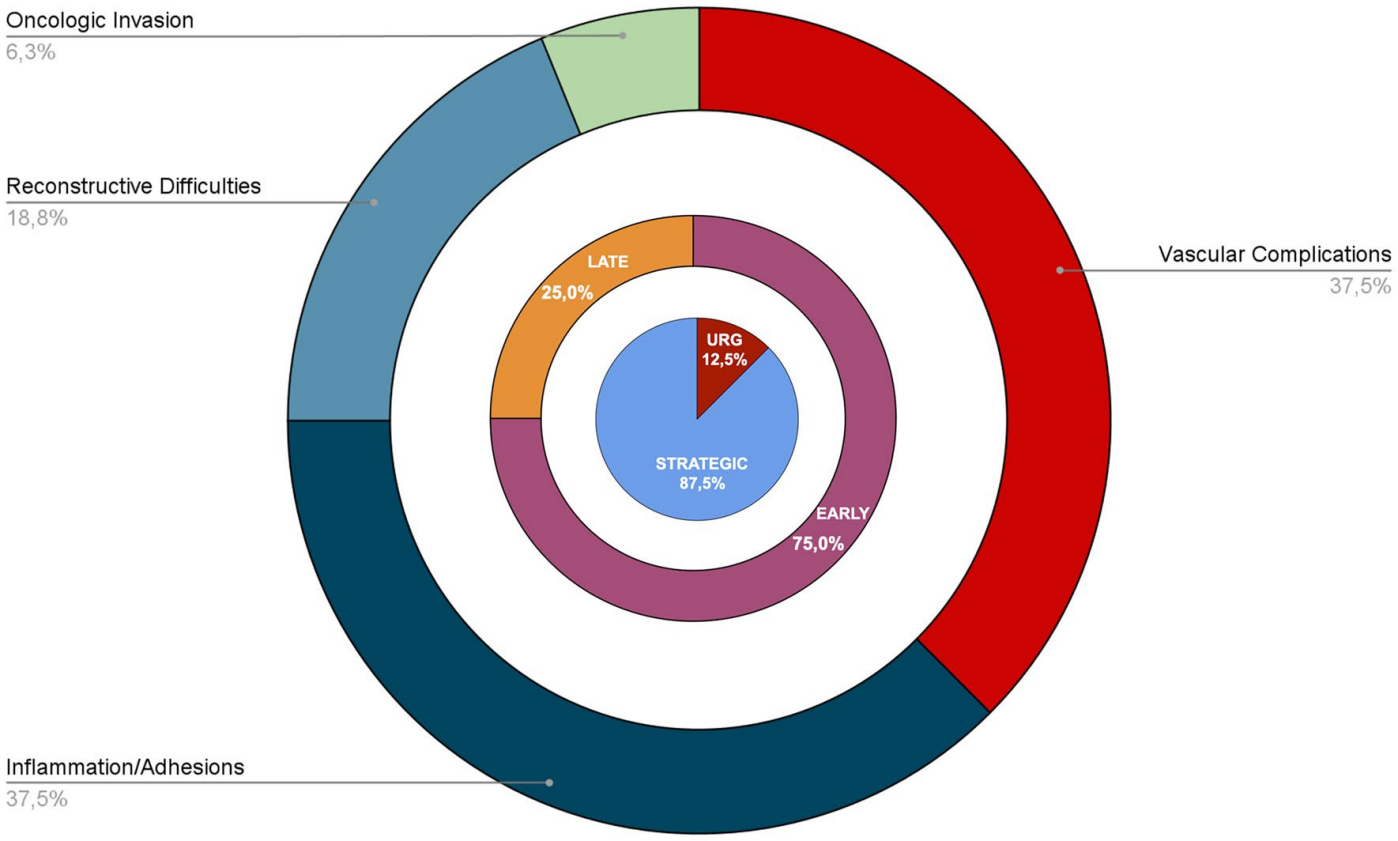
Reasons, timing, and intent of conversion during robotic pancreaticoduodenectomy. The outer ring illustrates the reasons for conversion, the middle ring the timing of conversion, and the inner ring the intent of conversion. Vascular complications and inflammatory or adhesive conditions represented the most frequent causes. Most conversions occurred early during the procedure (12/16, 75%) and were strategic (14/16, 87.5%), while only two cases required urgent conversion due to uncontrolled venous bleeding

To further characterize the mechanisms underlying conversion, operative reports were systematically reviewed and reasons for conversion were grouped into four predefined categories (Fig. 2).

Vascular complications represented one of the most frequent causes of conversion (6 of 16 cases), including portal or mesenteric venous injury, portal vein stenosis requiring resection, and bleeding at the gastroduodenal artery stump or portal anastomosis requiring open vascular control. Inflammatory or adhesive conditions accounted for six additional conversions, mainly related to dense peripancreatic inflammation, post-endoscopic retrograde cholangiopancreatography (ERCP) pancreatitis, or extensive peritumoral adhesions.

Technical or reconstructive considerations led to three conversions. In these cases, the decision was not related to limitations in robotic visualization but rather to concerns regarding safe reconstruction after technically demanding resections and in the presence of very small pancreatic ducts within soft glands. Finally, oncologic invasion precluding safe dissection was identified in one case.

Conversion timing and intent were further characterized descriptively. Among the 16 conversions, 12 (75%) occurred early in the procedure, before pancreatic transection or major vascular dissection, whereas 4 (25%) occurred later during the resection or reconstructive phase. With respect to intent, most conversions were strategic (14/16, 87.5%), performed in hemodynamically stable conditions due to technical complexity or unfavorable anatomy, while only two conversions (12.5%) were urgent and occurred in the setting of uncontrolled venous bleeding.

Descriptive stratification suggested less favorable postoperative outcomes in late conversions compared with early conversions, including longer operative time (median 605 vs 560 min) and longer hospital stay (median 33 vs 16.5 days). Postoperative complications were also more frequent in late conversions, including higher rates of hemorrhage (50% vs 25%), reoperation (25% vs 16.7%), and mortality (50% vs 16.7%). Urgent conversions were characterized by prolonged operative time, intraoperative transfusion, and venous resection in both cases, with one patient experiencing postoperative hemorrhage complicated by mesenteric ischemia leading to death at 30 days.

## Discussion

The present findings highlight important elements influencing when and why conversion occurs during RPD and how these events translate into perioperative outcomes. A key strength of this study is the identification of predictors that are recognizable preoperatively or very early during the operation. This aspect is particularly relevant in robotic pancreatic surgery, as early recognition of conversion-predisposing factors allows optimization of patient selection, operative planning, and team readiness. Importantly, the predictors identified in our analysis predominantly reflect anatomical and inflammatory complexity rather than surgeon inexperience, supporting the applicability of these findings across centers with varying robotic volumes.

In our experience, the conversion rate of 12.3% during RPD is consistent with contemporary robotic series, which generally report conversion rates between 5 and 13%, and remains substantially lower than those historically described for laparoscopic pancreaticoduodenectomy [2, 3, 16].

Beyond its occurrence, conversion should also be interpreted in terms of timing and intent. Large multicenter analyses, such as the European E-MIPS study by Löf et al., have highlighted the distinction between emergency conversions, often triggered by acute intraoperative events such as bleeding, and elective or strategic conversions undertaken in a controlled setting when operative conditions are unfavorable but stable [2]. In line with these observations, anticipating conversion risk based on preoperative imaging and clinical history facilitates deliberate, early conversion rather than late, reactive conversion after prolonged dissection, cumulative inflammation, or significant blood loss.

In selected situations in our series, conversion occurred during the reconstructive phase when concerns arose regarding the safety of pancreatic or biliary reconstruction after technically demanding resections. These circumstances did not reflect limitations of robotic visualization but rather a deliberate decision to perform reconstruction under optimal technical conditions. Within this framework, conversion should be regarded as appropriate surgical judgment rather than failure of the minimally invasive approach, a concept further supported by the low proportion of emergency conversions observed in the present robotic series (2 of 16).

### Key drivers of conversion: what can we anticipate?

In the multivariable model, vascular involvement requiring venous resection and periampullary tumor location remained independently associated with conversion, whereas a history of pancreatitis showed a borderline association after penalized adjustment.

Vascular involvement emerged as the strongest factor associated with conversion, highlighting the impact of anatomical complexity on intraoperative decision-making during RPD. In our analysis, vascular contact was defined preoperatively at initial staging, thereby reflecting anticipated technical difficulty rather than an intraoperative event. This distinction reinforces its relevance as a planning variable for case selection and operative strategy. However, although defined on pre-treatment imaging, vascular contact may also partially reflect treatment selection processes, including response to neoadjuvant therapy and subsequent case selection, and should therefore be interpreted with this potential influence in mind. The association between vascular involvement and conversion is consistent with findings from high-volume robotic series and multicenter analyses, including the study by Löf et al., in which vascular complexity and bleeding were leading drivers of conversion [2]. Similarly, robotic difficulty-scoring systems such as the PD-ROBOSCORE and the Tampa Difficulty Score have identified vascular involvement, venous resection, and bleeding-related parameters as markers of increased surgical complexity [17, 18]. These observations underscore the critical role of meticulous preoperative staging, particularly triphasic CT with dedicated vascular reconstructions, in identifying tumor-vessel relationships, venous abutment, caliber changes, or collateralization that may compromise the safety of robotic dissection.

Non-pancreatic tumor location, particularly distal bile duct and duodenal lesions, was also associated with a higher likelihood of conversion. These lesions are classically associated with a soft pancreatic gland and a small main pancreatic duct, a well-established anatomical setting linked to a challenging reconstruction [19, 20]. This situation contrasts with PDAC in which the resection phase may be more technically demanding while reconstruction is often facilitated by a firm gland and a dilated duct. In periampullary tumors, although resection is often considered technically favorable, surgeons may encounter greater reconstructive challenges related to gland texture and duct size. In addition, certain periampullary lesions may also present resection-related difficulty, particularly in the presence of local inflammation or complex dissection within the hepatoduodenal ligament, sometimes accentuated by prior endoscopic interventions such as biliary stenting or prosthesis placement. In selected intraoperative scenarios, the combination of these factors may increase overall procedural complexity and contribute to lowering the surgeon’s threshold for conversion when operative conditions become unfavorable.

Finally, a history of pancreatitis was included among the variables associated with conversion on univariable analysis, although it did not retain independent statistical significance after penalized adjustment and should therefore be interpreted cautiously. Pancreatitis is known to induce peripancreatic fibrosis, obliteration of tissue planes, and increased perivascular fragility, which may complicate dissection and increase the likelihood of conversion [21]. Moreover, recent radiologic studies have highlighted the role of pancreatic parenchymal biology and inflammation in shaping perioperative risk, supporting a potential link between pancreatic inflammatory status and operative complexity [22].

Taken together, these findings highlight clinically meaningful factors that may influence intraoperative decision-making during RPD. They should therefore be interpreted as situation-dependent determinants of conversion within a specific surgical environment, likely influenced by surgeon-dependent thresholds and individual decision-making strategies, rather than as universal or purely predictive risk factors. This supports the view that conversion during RPD reflects a nuanced intraoperative judgment rather than a strictly predictable event.

### Learning curve: how much does it matter?

The impact of surgical experience on outcomes in robotic pancreatic surgery has progressively shifted from a focus on technical feasibility to a broader understanding of how expertise influences case selection, intraoperative decision-making, and procedural safety. Nationwide data from the Dutch Pancreatic Cancer Group have demonstrated a steady increase in annual RPD volume with a relatively stable conversion rate, despite growing procedural complexity over time [23]. This pattern closely mirrors our findings, in which conversion numbers remained stable even as overall caseload increased (Fig. 1).

The learning curve of RPD is widely recognized as a stepwise process, progressing from feasibility to proficiency, followed by an advanced phase and eventual mastery (> 84 procedures) [24]. As reported by Napoli et al., surgeons operating in the advanced and mastery phases increasingly undertake complex cases, including patients with high fistula risk, prior neoadjuvant therapy, or vascular involvement, without a proportional increase in conversion or adverse outcomes [25]. This evolution reflects a transition from technical refinement to a deliberate and controlled expansion of robotic indications as experience accrues.

In our cohort, learning-curve status was not independently associated with conversion, suggesting that conversion was driven primarily by anatomical complexity and intraoperative findings rather than by lack of experience. This observation is consistent with a prior institutional analysis of the operating surgeon in this series, in which proficiency was achieved after the first 40 robotic procedures [10]. Within this framework, our data support the concept of a *second-phase learning curve* in RPD, whereby increasing experience enhances the surgeon’s ability to anticipate challenges, maintain stable conversion rates, and safely incorporate more complex patients into the robotic program.

It should also be noted that this robotic series represents the first 130 RPDs performed by a surgeon with extensive prior experience in open pancreatic surgery (> 500 open pancreaticoduodenectomies). In selected technically demanding situations, particularly during reconstruction after complex resections, conversion may therefore reflect a deliberate decision to perform the reconstructive phase in the operative setting with the greatest cumulative surgical experience and tactile feedback rather than a limitation of the robotic approach itself.

This provides essential context for interpreting the postoperative impact of conversion in a mature robotic practice. In parallel, increasing institutional access to the robotic platform during the study period facilitated broader integration of RPD into clinical practice, allowing the progressive inclusion of more technically complex cases.

### Does conversion really worsen outcomes?

Given the limited number of converted cases, postoperative outcomes were intentionally summarized using effect size measures rather than relying solely on hypothesis testing, in order to better convey the magnitude and direction of observed differences. In our cohort, conversion to an open approach was not associated with a clear increase in overall or major postoperative complication rates, nor with increased rates of clinically relevant POPF, biliary leak, delayed gastric emptying, and short-term mortality compared with fully robotic procedures. Although the biliary fistula rate observed in our cohort may appear slightly higher than that reported in some large contemporary series, where rates after pancreaticoduodenectomy generally range between approximately 4% and 10%, this finding should be interpreted cautiously given the limited number of events in this single-center cohort [15, 26]. Notably, converted cases were characterized by a significantly higher rate of intraoperative bleeding requiring transfusion, reflecting increased perioperative complexity rather than a direct worsening of postoperative morbidity.

By contrast, length of hospital stay and HDU stay were significantly longer in the converted group, indicating that conversion primarily impacts postoperative resource utilization rather than the severity of postoperative morbidity.

The absence of significant differences in overall or major postoperative complications between converted and non-converted cases should therefore be interpreted in light of the organizational and procedural context in which these operations were performed, rather than as evidence of equivalent surgical complexity or negligible clinical impact. In high-volume settings, accumulated experience often translates less into a reduction in complication incidence and more into improved anticipation, early recognition, and structured management of adverse events [27]. This concept is well illustrated by the nationwide implementation of the PORSCH algorithm, which promotes standardized postoperative surveillance and a step-up, minimally invasive management strategy for POPF [28]. The so-called PORSCH effect refers to the observation that earlier detection and structured management of complications are associated with higher identification of grade B fistulas and increased use of interventional treatments, while simultaneously reducing the occurrence of grade C fistulas, failure to rescue, and postoperative mortality [29]. In this context, the PORSCH experience provides a useful conceptual framework for interpreting our findings. Within this framework, it is plausible that robust perioperative pathways, early decision-making, and timely intervention attenuated the downstream clinical consequences of conversion in our series, preserving short-term outcomes despite the higher anatomical and procedural complexity of converted cases.

Our results contrast with larger series such as that of Slavin et al., in which unplanned conversions during RPD were associated with substantially higher major complication rates, prolonged ICU stays, and increased mortality, largely driven by intraoperative hemorrhage and physiological instability [3]. This divergence likely reflects differences in the timing and intent of conversion. In our experience, conversion was frequently anticipated based on preoperative imaging or early intraoperative findings rather than triggered by uncontrolled bleeding or hemodynamic deterioration, with emergency conversions accounting for only 2 of 16 cases.

These results suggest that the context, timing, and intent of conversion may be more clinically meaningful than conversion itself. When anticipated and performed early within structured perioperative pathways, conversion may preserve postoperative outcomes, while being associated with increased hospital resource utilization.

### Limitations and future directions

This study has several limitations that should be acknowledged. First, the limited sample size (130 patients, including 16 conversions) reduced statistical power, resulting in wide confidence intervals. Rather than representing a methodological weakness, these reflect the inherent uncertainty associated with sparse event data and were deliberately retained to avoid overinterpretation. Accordingly, Firth penalized logistic regression was used as a bias-reduction method suitable for small or sparse binary datasets with low event-per-variable ratios and potential separation [30]. In this context, the magnitude of the observed effect estimates, particularly the large odds ratios, should be interpreted with caution. Second, the retrospective, single-center design is subject to selection biases and unmeasured confounding. Third, all procedures were performed by a single high-volume surgeon, enhancing internal consistency but reducing generalizability, as conversion thresholds and operative strategies are inherently influenced by individual experience and intraoperative decision-making, which may limit the applicability of these findings to other settings.

Another limitation is the absence of a validated conversion risk score for RPD. While predictive tools exist for minimally invasive distal pancreatectomy, no comparable framework is currently available for RPD, despite its higher technical complexity. In this context, our analysis should be interpreted as exploratory and hypothesis-generating rather than definitive risk prediction. The complementary reporting of odds ratios, confidence intervals, and absolute risks was deliberately chosen to provide a balanced and clinically interpretable representation of risk in a small cohort.

Future research should evaluate whether advanced imaging, intraoperative data, or video-based metrics can enhance prediction models beyond standard clinical factors. Clarifying how surgeon thresholds, team dynamics, and institutional pathways influence conversion decisions, as well as differentiating early planned from late reactive conversion, may further guide strategies to improve safety and outcomes.

## Conclusion

In this tertiary-center experience, conversion during RPD was mainly influenced by preoperatively identifiable anatomical and disease-related factors, such as vascular involvement and periampullary tumor location, rather than by surgeon inexperience. When anticipated and performed in a controlled manner within a mature robotic program, conversion was not associated with a clear increase in major postoperative morbidity, although it resulted in longer hospital and HDU stay. These findings support the view that conversion during RPD should be regarded as a deliberate, context-dependent, safety-driven, intraoperative decision rather than a failure of minimally invasive surgery. Future multicenter studies are warranted to validate dedicated conversion risk models and to further refine patient selection and operative planning.
